# Ensemble signal detection reveals target-specific temporal risk of hypokalemia after immune checkpoint inhibition: a translational pharmacology framework

**DOI:** 10.3389/fphar.2026.1960328

**Published:** 2026-10-01

**Authors:** Shengwang Chen, Ziyi Dai, Zihan Guo, Ziyi Li, Ruiqi Liu, Xuewen Jiang

**Affiliations:** 1 Department of Neurosurgery, The Third Hospital of Xiamen, Xiamen City, Fujian, China; 2 The Second Clinical College of China Medical University, Shenyang, China; 3 Dalian Medical University, Dalian, China; 4 The First Clinical College of China Medical University, Shenyang, China; 5 Department of Neurosurgery, Xiamen Xinglin Hospital, Xiamen, China

**Keywords:** computational pharmacovigilance, ensemble signal detection, hypokalemia, immune checkpoint inhibitors, precision medicine, real-world evidence, temporal risk pattern, translational pharmacology

## Abstract

**Introduction:**

Immune checkpoint inhibitors (ICIs) have revolutionized cancer treatment, but traditional pharmacovigilance lacks a translational computational framework capable of resolving target-specific nonlinear temporal risks of adverse events such as hypokalemia.

**Methods:**

We integrated 3,243 ICI-related hypokalemia reports from the FDA Adverse Event Reporting System (FAERS) and VigiBase (2014–2024). An ensemble signal-detection strategy cross-validated four disproportionality algorithms (Reporting Odds Ratio [ROR], Proportional Reporting Ratio [PRR], Empirical Bayesian Geometric Mean [EBGM], and Information Component [IC]). Flexible nonlinear survival analysis was performed using restricted cubic spline (RCS) Cox regression to model time-to-event dynamics.

**Results:**

Programmed Death-Ligand 1 (PD-L1) inhibitors triggered the strongest signal (ROR = 3.20, 95% CI: 2.95–3.48), with ultra-early onset (median 23 days) and the highest case fatality rate (23.7%). An asymmetric biphasic mortality pattern showed a nadir at 60 days: early-onset (≤30 days) hypokalemia peaked at a hazard ratio (HR) of ∼1.9, whereas late-onset (>90 days) risk gradually rose to HR ∼1.8 within 350 days. Programmed Death-1 (PD-1) inhibitors significantly increased severe hypokalemia risk (odds ratio [OR] = 28.32), with male predominance across PD-1 and CTLA-4 subgroups.

**Discussion:**

This interpretable ensemble framework reveals target-specific temporal toxicity signatures from multi-source real-world data, providing a computationally grounded translational pharmacology foundation for precision electrolyte monitoring and mechanism-informed safety prediction.

## 1 Introduction

Combining artificial intelligence and computational methods enables pharmacovigilance to move beyond simple passive signal detection and transform into a predictive, mechanism-informed safety science. Traditional disproportionality analysis is less capable than machine learning methods in handling high-dimensional and temporally complex real-world data; machine learning approaches—including ensemble methods and nonlinear survival modeling—can more effectively process such data (Algarvio et al., 2025; Nagar et al., 2025; Kompa et al., 2022).

However, current computational pharmacovigilance still faces critical limitations: black-box predictions often lack clinical interpretability; most models ignore the nonlinear temporal dynamics between drug exposure, adverse event onset, and mortality; and few studies connect computational signals to target-specific inflammatory mechanisms that can be validated by multi-omics. Consequently, there remains an unmet need for interpretable computational frameworks that integrate multi-source real-world data with mechanism-oriented hypotheses (Suryadevara et al., 2026; Tjoa and Guan, 2021).

Immune checkpoint inhibitors (ICIs) restore the body’s immune killing function against tumor cells by blocking the interaction between inhibitory receptors on T cell surfaces (such as Programmed Cell death Protein 1 (PD-1), Programmed death-ligand 1 (PD-L1), Cytotoxic T-Lymphocyte-associated Protein 4 (CTLA-4), Lymphocyte-Activation Gene 3 (LAG-3)) and their ligands, thereby relieving immunosuppression of T cells in the tumor microenvironment (Sharma et al., 2023; Chen et al., 2012; Topalian et al., 2015). After the non-specific immune system is activated, it will have an impact on multiple organ systems and then cause immune-related adverse events (irAEs) (Martins et al., 2019; Postow et al., 2018). These toxicities manifest as disruption of peripheral immune tolerance, abnormal activation of T cells and cytokine release, which indicates that ICI adverse reactions are caused by organ-specific inflammatory damage rather than uniform off-target toxicity.

Hypokalemia is a clinically significant yet under-characterized immune-related adverse event (irAE). Beyond immediate cardiac consequences, severe hypokalemia can also cause neuromuscular dysfunction, specifically characterized by progressive weakness, fatigue, and, in severe cases, paralysis of the respiratory muscles, which may lead to acute respiratory failure requiring intensive care (Rajaram et al., 2025; Unwin et al., 2011). These manifestations are particularly relevant in cancer patients receiving immunotherapy, among whom fatigue is the most commonly reported symptom and greatly interferes with quality of life (Mamoor et al., 2020). The differential expression of ICI targets in non-tumor tissues exacerbates vulnerability. PD-L1 is constitutively expressed on renal tubular epithelial cells, which predisposes to T-cell-mediated tubular injury and cytokine-driven electrolyte wasting upon blockade (Sprangers et al., 2022; Tian et al., 2022), whereas CTLA-4 is expressed on pituitary cells and its blockade induces delayed T-cell-mediated inflammation disrupting hypothalamic-pituitary-adrenal axis function (Faje et al., 2014; Cui et al., 2022). Target-specific inflammatory mechanisms show differences in the time patterns of electrolyte disturbances, but current guidelines recommend unified laboratory monitoring without target-specific guidance (Puzanov et al., 2017).

In this study, we propose a hybrid computational framework that integrates ensemble signal detection with cross-validated disproportionality algorithms, nonlinear machine learning-informed survival analysis via restricted cubic spline Cox modeling, and an interpretable clinical prioritization algorithm to dissect the target-specific temporal risk patterns of ICI-associated hypokalemia. By linking these computational phenotypes to organ-specific inflammatory mechanisms, we establish testable hypotheses for future multi-omics integration (Fusaroli et al., 2024).

## 2 Materials and methods

### 2.1 Data sources and acquisition

This study analyzed de-identified records from the FDA Adverse Event Reporting System (FAERS) and WHO VigiBase (2014–2024) (Lindquist, 2008; Potter et al., 2025). Cases were coded using MedDRA version 25.0. Inclusion criteria: ICI as primary suspected agent, hypokalemia or related electrolyte disturbances, complete temporal data, age ≥18 years.

### 2.2 Drug and event definitions

CIs included PD-1 inhibitors (cemiplimab, nivolumab, pembrolizumab), PD-L1 inhibitors (atezolizumab, avelumab, durvalumab), CTLA-4 inhibitors (ipilimumab, tremelimumab), and LAG-3 inhibitor (relatlimab). Events encompassed hypokalemia, hypokalemic syndrome, decreased serum potassium, abnormal serum potassium, electrocardiogram U-wave abnormalities, and hypomagnesemia.

### 2.3 Ensemble signal detection via multi-algorithm cross-validation

To enhance signal robustness and overcome the limitations of a single algorithm, we employed an ensemble method integrating four disproportionality metrics (ROR, PRR, EBGM, IC). This approach is analogous to bagging strategies in machine learning, where multiple weak learners are combined to reduce both false-positive and false-negative rates (Jiao et al., 2024).

Analyses followed READUS-PV guidelines (Fusaroli et al., 2024). A completed READUS-PV checklist is provided as Supplementary Information. Our disproportionality methods (ROR, PRR, EBGM, IC) were used for cross-validation across frequency-based and Bayesian dimensions to reduce false positives and negatives. The specific criteria for each method are as follows: lower limit of 95% Confidence Interval (CI) for ROR > 1 and number of reports ≥ 3; PRR ≥ 2, $x^{2}$ ≥ 4, and number of reports ≥ 3; EBGM_05_ > 1; IC_025_ > 0 (Bate et al., 1998; Dumouchel, 1999; Evans et al., 2001; van Puijenbroek et al., 2002).

We selected ROR and PRR as representative frequentist measures, and EBGM and IC as representative Bayesian metrics, to ensure coverage across both statistical paradigms. This four-algorithm configuration balances computational accessibility with robust cross-validation, and aligns with the READUS-PV reporting guidelines (Fusaroli et al., 2024). Alternative Bayesian shrinkage approaches such as MGPS and BCPNN were not included because the selected metrics already span the frequency-Bayesian spectrum and are sufficient for consensus-based signal triangulation in this dataset.

### 2.4 Statistical analysis

Descriptive statistics used median (IQR) for continuous variables and frequencies for categorical variables. Group comparisons employed chi-square, Fisher’s exact, Mann-Whitney U, or Kruskal–Wallis tests. Two-tailed P < 0.05 indicated significance. Sample sizes (FAERS: 2,143; VigiBase: 1,100) achieved >80% power to detect ROR ≥ 2 (α = 0.05).

### 2.5 Nonlinear machine learning-based survival analysis

The traditional Cox proportional hazards model assumes a linear association between continuous predictor variables and the log-hazard. We employed restricted cubic spline (RCS) Cox modeling, which is a flexible nonlinear extension of survival analysis, to capture complex and biologically plausible associations from disease onset to death (Schuster et al., 2022).

The RCS Cox models are used to explore the nonlinear relationship between time to onset (TTO) and mortality. Four knots are set at the 5th, 35th, 65th, and 95th percentiles, following Harrell’s recommendation of 3–5 knots for adequate flexibility without overfitting (Schuster et al., 2022). While we did not perform exhaustive sensitivity analyses, exploratory specifications with 3 and 5 knots yielded consistent nadir positions and curve shapes, supporting the robustness of the observed asymmetric pattern. The models are adjusted for age, sex, and reporting year, and the likelihood ratio test is used to evaluate the significance of nonlinearity.

### 2.6 Interpretable clinical decision algorithm for risk stratification

Inspired by explainable AI principles, we developed a transparent, rule-based priority scoring system where each dimension represents a clinically interpretable variable (Tjoa and Guan, 2021; Rudin, 2019).

We adapted a four-dimensional priority scoring system from Cecco et al. (originally developed for ADC toxicities) to evaluate ICI-associated hypokalemia risk (score range: 0–8) (Cecco et al., 2024). Dimension 1 (Clinical relevance): Designated Medical Event (DME) = 2 points, Important Medical Event (IME) = 1 point, none = 0 (per European Medicines Agency [EMA] classification). Dimension 2 (Reporting rate): greater than 10% = 2 points, 1%–10% = 1 point, <1% = 0. Dimension 3 (Association stability): 4 positive algorithms = 2 points, 2–3 = 1 point, <2 = 0. Dimension 4 (Case fatality rate): >50% = 2 points, 25%–50% = 1 point, <25% = 0. Priority thresholds: high (6–8 points, consider drug discontinuation), medium (3–5 points, enhanced monitoring), low (0–2 points, routine monitoring). Detailed scoring is provided in Figure 1D.

**FIGURE 1:**
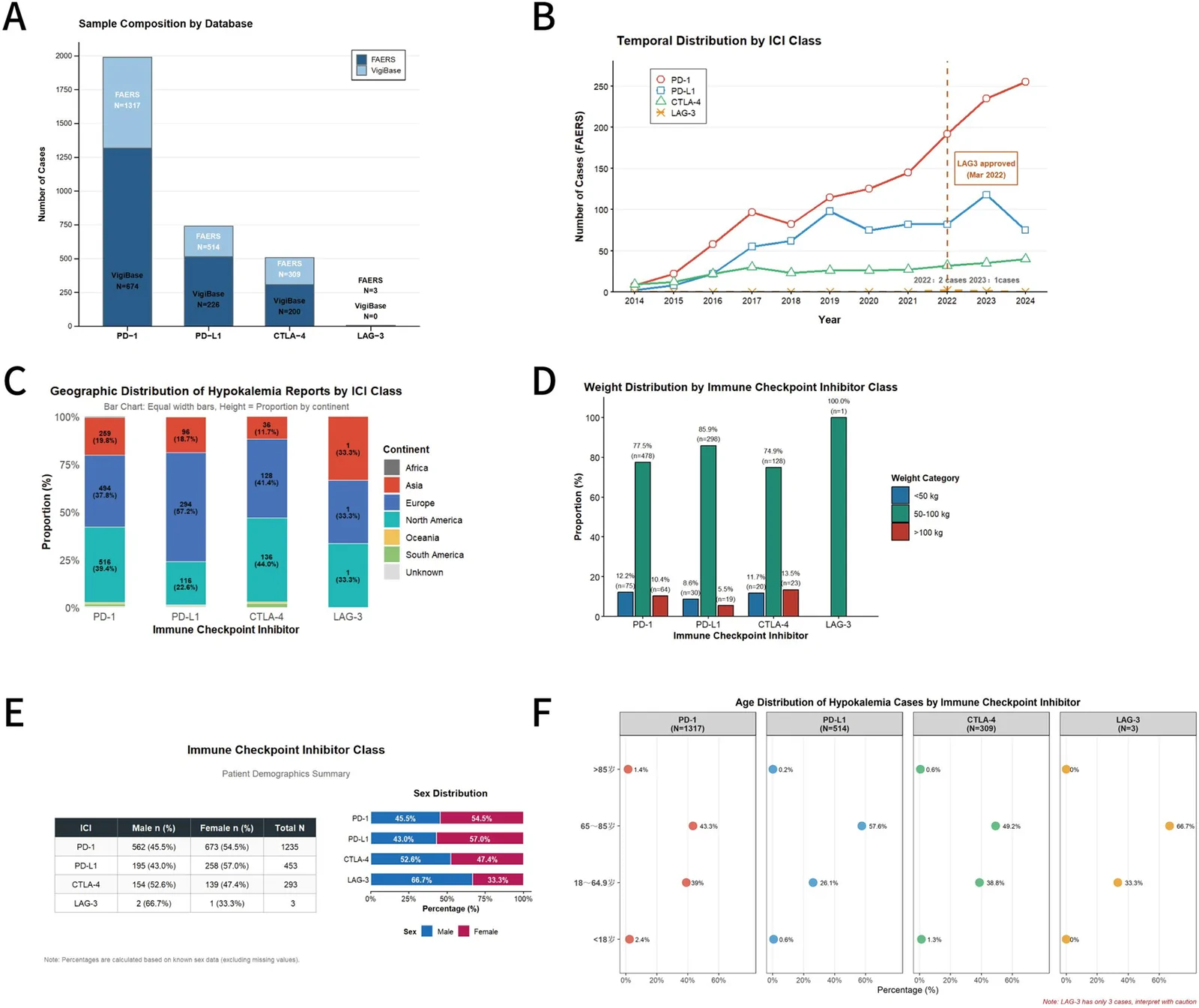
Baseline Characteristics of Immune Checkpoint Inhibitor (ICI)-Associated Hypokalemia. **(A)** Sample Composition by Database. Stacked bar chart displaying the distribution of hypokalemia reports across different ICI classes (PD-1 inhibitors, PD-L1 inhibitors, CTLA-4 inhibitors, and LAG-3 inhibitors) in FAERS and VigiBase databases. Dark blue represents FAERS data, light blue represents VigiBase data. Absolute report numbers for each ICI category within each database are shown inside the bars. **(B)** Temporal Distribution Trends by ICI Class. The x-axis represents the reporting year. The y-axis represents the number of adverse event reports. Lines of different colors represent different inhibitors. Red represents PD-1 inhibitors. Blue represents PD-L1 inhibitors. Green represents CTLA-4 inhibitors. The orange dashed line represents LAG-3 inhibitors. A dashed line indicates the date of approval for LAG-3 inhibitors. That date is March 2022. Each data point is labeled. There were 2 cases in 2022. There was 1 case in 2023. **(C)** Geographic Distribution of Hypokalemia Reports by ICI Class. It illustrates the distribution of reported cases of hypokalemia, broken down by continent. The chart covers various categories of immunosuppressive drugs. All columns are the same width. The height of each column indicates the proportion of reports from each continent. The colors represent different regions. Red represents Asia. Blue represents Europe. Cyan represents North America. Green represents South America. Yellow represents Oceania. Gray represents Africa. Each column has a label. The labels show the number of reports. They also display the percentage for each continent. **(D)** Body Weight Distribution by ICI Class. It shows the distribution of patients’ body weight. This distribution covers different categories of ICI drugs. Body weight is divided into three groups. One group weigh less than 50 kg and is shown in blue. One group of patients with body weight between 50 and 100 kg was given a green label. The last group of patients with body weighs over 100 kg was given a red label. Percentages and sample sizes for each weight category are labeled above the bars. **(E)** Patient Demographics Summary. Composite chart containing two components. Left panel: Summary table of gender distribution by ICI class, showing male and female case counts and percentages. Right panel: Horizontal bar chart displaying gender proportion distribution for each ICI drug class, with blue representing male and red representing female. **(F)** Age Distribution of Hypokalemia Cases by ICI Class. Dot plot showing age distribution of patients across different ICI drug classes. Age is categorized into four groups: <18 years, 18–64.9 years, 65–85 years, and >85 years. The X-axis represents percentage, and the Y-axis represents age groups. Different colored dots represent different ICI classes, with percentages labeled for each age group. Note: LAG-3 inhibitors had only 3 cases, requiring cautious interpretation.

## 3 Results

### 3.1 Baseline characteristics

A total of 2,143 reports of hypokalemia using ICIs were collected from FAERS, and 1,100 reports were collected from VigiBase. Figure 2A shows the distribution of drugs in the sample. The proportion of PD-1 inhibitors was relatively high (61.5% in FAERS, with 1,317 out of 2,143 cases; 61.3% in VigiBase, with a quantity of 674/1,100). PD-L1 inhibitors were behind (24.0% in FAERS; 20.5% in VigiBase). The proportion of CTLA-4 inhibitors was 14.4% (FAERS) and 18.2% (VigiBase). The number of reports for LAG-3 inhibitors was relatively small, with only 3 cases in FAERS.

**FIGURE 2:**
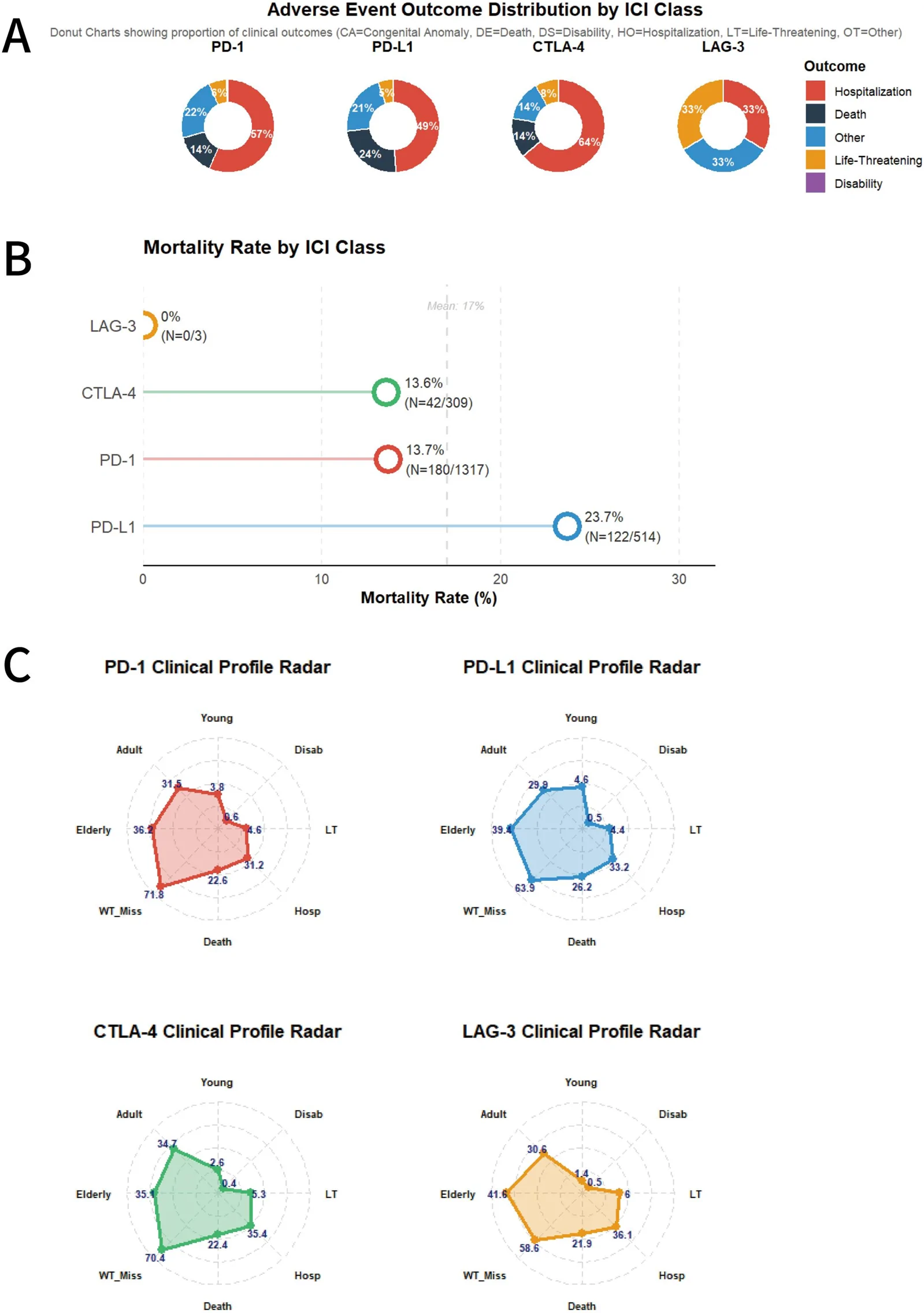
Clinical Outcomes and Mortality Analysis of Immune Checkpoint Inhibitor (ICI)-Associated Hypokalemia (FAERS Database). **(A)** Distribution of Adverse Event Outcomes by ICI Class. Donut charts displaying the clinical outcome composition of hypokalemia reports associated with different ICI drugs in the FAERS database. From left to right: PD-1 inhibitors (N = 1,317), PD-L1 inhibitors (N = 514), CTLA-4 inhibitors (N = 309), and LAG-3 inhibitors (N = 3). Color coding: red (hospitalization, HO), dark gray (death, DE), blue (other, OT), orange (life-threatening, LT), purple (disability, DS), cyan (congenital anomaly, CA). Report counts and percentages for each outcome are labeled, with only labels for proportions >3% displayed. **(B)** Mortality Rates by ICI Class. Lollipop chart showing case fatality rates (number of deaths/total reports) for hypokalemia associated with different ICI drug classes. The X-axis represents mortality rate (%), and the Y-axis represents ICI class. PD-L1 inhibitors demonstrate the highest mortality rate (23.7%, 122/514), significantly higher than PD-1 inhibitors (13.7%, 180/1,317) and CTLA-4 inhibitors (13.6%, 42/309) (P < 0.001). LAG-3 inhibitors have no death reports (0%, 0/3). Dashed line indicates the average mortality rate (17%). **(C)** Clinical Characteristics Radar Charts. Four radar charts displaying multi-dimensional clinical characteristic distributions for patients with hypokalemia associated with PD-1, PD-L1, CTLA-4, and LAG-3 inhibitors. Eight dimensions include: Death, Hospitalization (Hosp), Life-threatening (LT), Disability (Disab), Young (<18 years), Adult (18–64.9 years), Elderly (65–85 years), and Missing Weight (WT_Miss). Values represent percentages of each dimension within total reports. Color coding: PD-1 (red), PD-L1 (blue), CTLA-4 (green), LAG-3 (orange) Note: LAG-3 inhibitors had only 3 cases, requiring cautious interpretation.

The temporal trend in Figure 2B shows a steady upward trend in cases from 2014 to 2024. The number of reports of PD-1 inhibitors was less than 10 cases in 2014 and more than 250 cases in 2024. Relevant reports appeared less than 1 year after the approval of LAG-3 inhibitors.

Figure 2C shows the geographical distribution. The United States was the largest source of reports, among which the proportion of PD-1 is 35.2%, the proportion of PD-L1 is 19.3%, and the proportion of CTLA-4 is 40.8%. Asia (mainly Japan) and Europe (Germany, France, Italy) belong to secondary sources. The proportion of PD-1 inhibitor reports in Asia is 19.8%, which is higher than that of PD-L1 (18.7%) and CTLA-4 (11%).

Figure 2D shows that the majority of patients weighed between 50 and 100 kg (PD-1: 77.5%, PD-L1: 85.9%, CTLA-4: 74.9%). Among patients with a body weight exceeding 100 kg, the proportion of PD-L1 inhibitors (5.5%) is lower than that of the other two drug types. The gender distribution in Figure 2E shows that the proportion of female patients corresponding to PD-1 and PD-L1 inhibitors was relatively higher, which were 54.5% and 57.0% respectively. While the proportion of male patients corresponding to CTLA-4 and LAG-3 inhibitors was relatively higher, which were 52.6% and 66% respectively. The 65–85-year-old group is the most common in the age distribution in Figure 2F. Among LAG-3 inhibitors, 66.7% of elderly patients should be interpreted with caution because there were only three such patients in this drug category.

### 3.2 Analysis of clinical outcomes and mortality rates

The hypokalemia induced by immune checkpoints is mostly severe. Figure 3A pie chart shows the distribution of outcomes: hospitalization accounted for 57% of PD-1 cases, 49% of PD-L1 cases, and 64% of CTLA-4 cases. The mortality rates of PD-1 and CTLA-4 inhibitors are similar. The mortality rate of PD-L1 inhibitors is 23.7%, which was higher than that of PD-1 (13.7%) and CTLA-4 (13.6%) inhibitors.

**FIGURE 3:**
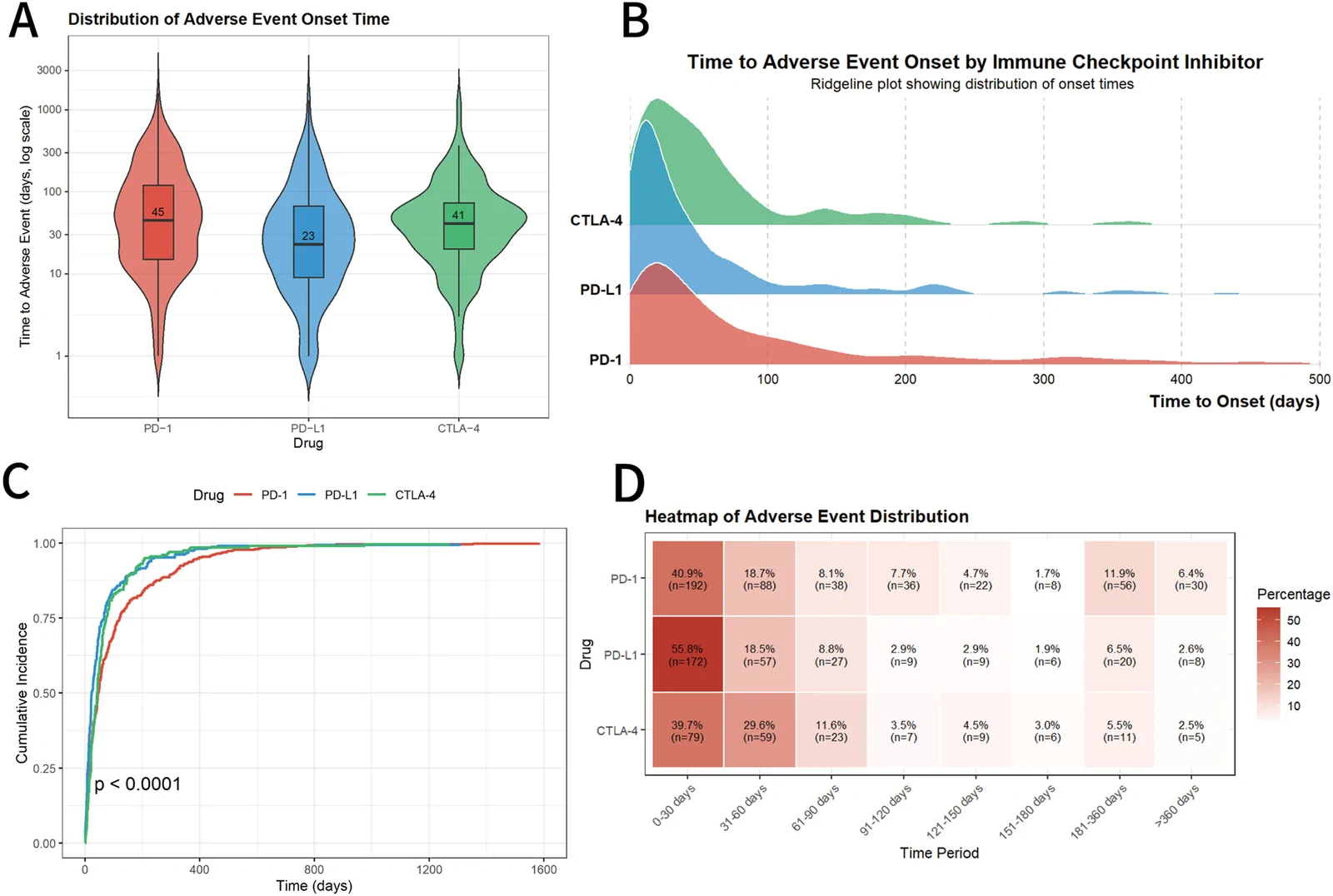
Time-to-Onset Patterns and Cumulative Incidence of Immune Checkpoint Inhibitor (ICI)-Associated Hypokalemia (FAERS Database). **(A)** Distribution of Adverse Event Time-to-Onset. Violin plots overlaid with box plots showing the time-to-onset distribution of hypokalemia associated with PD-1 inhibitors (red, N = 470), PD-L1 inhibitors (blue, N = 308), and CTLA-4 inhibitors (green, N = 199). The Y-axis is on a logarithmic scale (log scale), ranging 1–3,000 days. Box plots display median time-to-onset: 45 days for PD-1, 23 days for PD-L1, and 41 days for CTLA-4. LAG-3 inhibitors were excluded due to insufficient sample size (only 2 cases). **(B)** Time-to-Onset of Adverse Events by ICI Class. Ridgeline plot displaying density distributions of hypokalemia time-to-onset for three ICI drugs. The X-axis represents time-to-onset (days), and the Y-axis from top to bottom represents CTLA-4 (green), PD-L1 (blue), and PD-1 (red). Curve height indicates probability density, showing PD-L1 inhibitors have a significant early peak (0–30 days) with an “ultra-early burst” pattern; CTLA-4 and PD-1 inhibitors show relatively dispersed distributions. **(C)** Cumulative Incidence Curves. Kaplan-Meier curves showing cumulative incidence of hypokalemia over time for different ICI drug classes. The X-axis represents time (days), and the Y-axis represents cumulative incidence. We used different colors to distinguish between the various inhibitors. PD-1 is shown in red, PD-L1 in blue, and CTLA-4 in green. The P-value from the Log-rank test is less than 0.0001. This indicates a significant difference among the three groups. CTLA-4 and PD-1 show a rapid increase in the early stages, while PD-L1 exhibits a steady cumulative trend. **(D)** Heatmap of Adverse Event Distribution. Heatmap displaying percentage distribution of hypokalemia reports across different time windows for three ICI drugs. Time windows: 0–30 days, 31–60 days, 61–90 days, 91–120 days, 121–150 days, 151–180 days, 181–360 days, >360 days. Color intensity represents percentage magnitude (dark red >50%, white <10%). Each cell is labeled with percentage and absolute case count (n). PD-L1 shows the highest proportion in 0–30 days (55.8%, 172/308), presenting a “single-peak dominant” pattern; CTLA-4 shows a secondary peak at 31–60 days (29.6%).

The radar chart in Figure 3C presents eight aspects of clinical differences. PD-1 inhibitors perform prominently in weight loss (71%). The proportion of elderly patients was 36.2%. The proportion of weight loss in the PD-L1 inhibitor group is 33.2%, and the proportion of elderly patients was 39.4%. CTLA-4 inhibitors have weight loss (70.4%) and weight gain (35.4%) cases. The sample size of LAG-3 inhibitors is small. Its radar chart shows an irregular shape, and the fluctuations between dimensions were obvious.

### 3.3 Time-to-onset patterns and cumulative risk

977 reports are selected from the FAERS database, all with complete time data (CTLA-4: 199; PD-1: 470; PD-L1: 308). Reports with missing dates and calculation errors are excluded. The sample size of LAG-3 inhibitor was small; only two reports had statistically measurable time data, which were not included in the time curve analysis. The median time-to-onsets of different drugs in Figure 4A are quite different. PD - L1 inhibitors exhibited the shortest median time - to - onset, and then that of CTLA-4 inhibitor (41 days). The duration of PD-1 inhibitor was 45 days. The distribution of PD-L1 is at a medium-low level and relatively concentrated in time. The distribution of CTLA-4 and PD-1 is more dispersed, with a tailing phenomenon.

**FIGURE 4:**
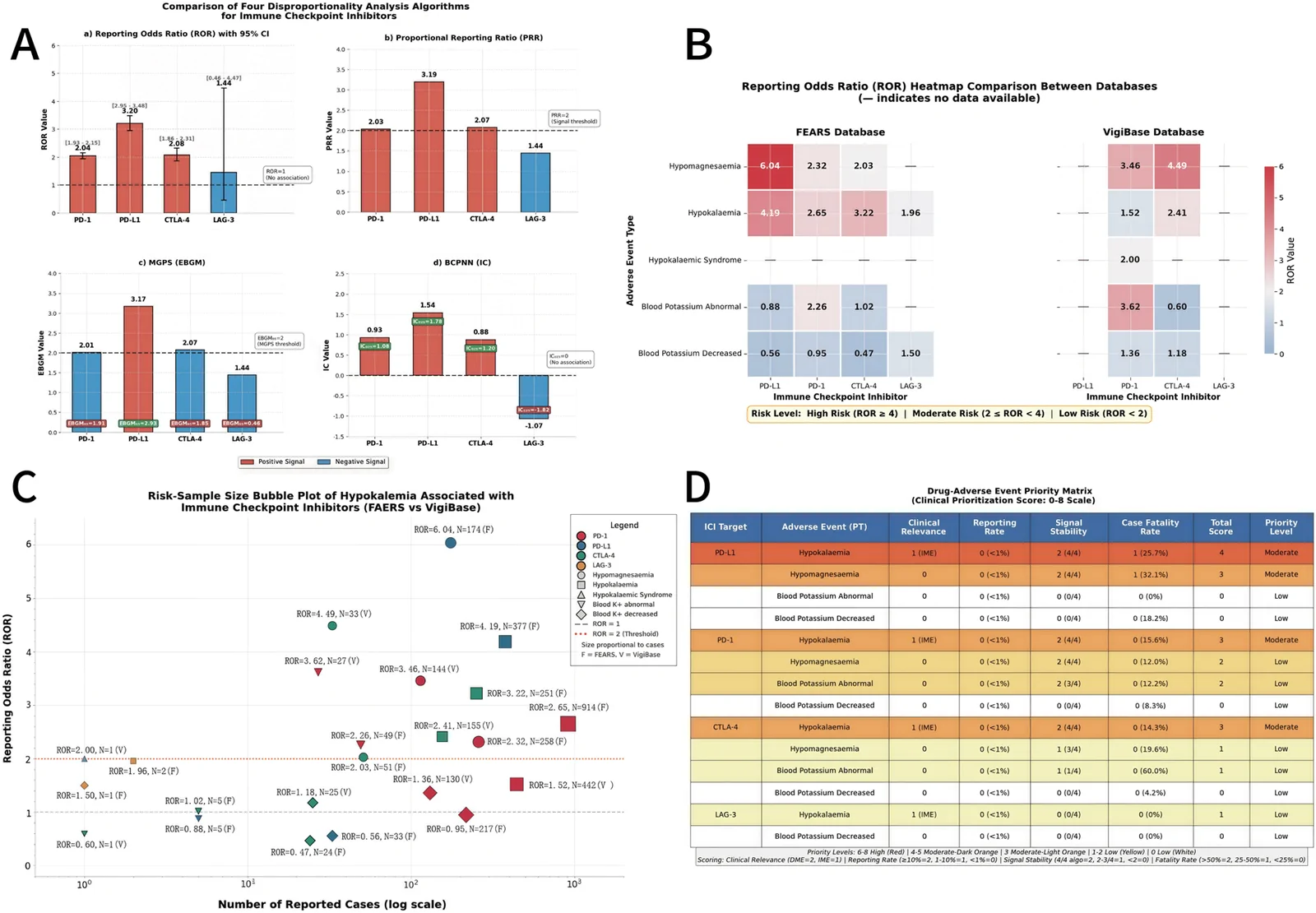
Pharmacovigilance Signal Strength and Clinical Priority Stratification of immune checkpoint inhibitor (ICI)-Associated Electrolyte Disorders. **(A)** Multi-Algorithm Signal Comparison. Bar chart comparing hypokalemia signal results across four disproportionality analysis algorithms (ROR, PRR, EBGM, IC) in four ICI drug classes. Error bars represent 95% confidence or credible intervals. Horizontal dashed line indicates signal threshold (lower limit of 95% CI > 1 or IC025 > 0). **(B)** Cross-Database ROR Heatmap Comparison. Heatmap displaying ROR values for different ICI drug classes across specific electrolyte disorder preferred terms (PTs) in FAERS (left) and VigiBase (right) databases. Color intensity represents signal strength (red: higher ROR, blue: lower ROR). PTs include: hypomagnesemia, hypokalemia, blood potassium decreased, blood potassium abnormal, and electrocardiogram U-wave abnormal. **(C)** Risk-Sample Size Bubble Plot. Bubble plot showing the relationship between electrolyte disorder PT signal strength (ROR, y-axis) and report count (x-axis, logarithmic scale). Bubble size is proportional to reported case numbers; bubble color indicates ICI type (red = PD-1, blue = PD-L1, green = CTLA-4, orange = LAG-3). Reference line indicates ROR = 1 (no signal). **(D)** Drug-Adverse Event Priority Matrix. Four-dimensional priority scoring matrix adapted from Cecco et al. (2024), evaluating ICI-associated electrolyte disorders based on: (1) clinical relevance (DME/IME classification), (2) reporting rate, (3) signal stability (consistency across 4 algorithms), and (4) case fatality rate. Color coding indicates priority levels: dark orange (4 points, medium-high priority), light orange (3 points, medium priority), yellow (2 points), light yellow (1 point), white (0 points). No high-priority events requiring immediate drug discontinuation (6–8 points, red zone) were identified. DME = Designated Medical Event; IME = Important Medical Event. Note: Signal strength and mortality in Figures 1C,D are presented at the PT level and may differ from Standardised MedDRA Query (SMQ)-level summary statistics shown elsewhere (e.g., Figures 3B, 1A).

The contour plots such as Figure 4B show different situations of density distribution. The PD-L1 inhibitor forms a sharp peak from 0 to 30 days, showing a “super-early surge” form. The distribution of CTLA-4 and PD-1 is relatively average, and the peaks appear relatively late. There were significant differences among these three groups (Log-rank P < 0.0001).

The heatmap in Figure 4D allows for a more precise analysis of the time window. The PD-L1 inhibitor exhibits a “single-peak dominant” pattern. 55.8% (172/308) of events occurred within 0–30 days. CTLA-4 inhibitors exhibit a “double-peaked sustained” pattern, with the second peak occurring between 31 and 60 days (29.6%). PD-1 inhibitors have the widest temporal distribution, with 6.4% of events occurring after 360 days.

### 3.4 Statistical association strength and cross-database validation

Figure 1A demonstrates the strongest association for PD-L1 inhibitors (ROR = 3.20, 95% CI: 2.95–3.48; PRR = 3.19; EBGM = 3.17; IC = 1.54), with all four algorithms exceeding thresholds (lower 95% CI of ROR >1 or IC_025_ > 0). CTLA-4 (ROR = 2.08) and PD-1 (ROR = 2.04) associations are moderate, while LAG-3 showed no significant association (95% CI of ROR spanned 1, EBGM_05_ < 1, IC < 0).

The cross-database heatmap in Figure 1B reveals heterogeneity at the PT level. The hypomagnesemia association for PD-L1 inhibitors in FAERS (ROR = 6.04) is 1.4 times higher than that for core diagnosis hypokalemia (ROR = 4.19); in VigiBase, the hypomagnesemia association for PD-1 inhibitors (ROR = 4.49) surpassed that for PD-L1 inhibitors (ROR = 3.46). Reduced serum potassium and abnormal serum potassium associations are generally weak, while ECG U wave associations failed to form stable associations due to sparse reporting.

The risk-sample size bubble chart in Figure 1C positions each PT’s clinical significance coordinates. PD-L1 hypomagnesemia (ROR = 6.04, N = 174) resides in the “high association-medium sample” quadrant; PD-1 hypokalemia (ROR = 2.65, N = 914) resides in the “high sample-low association” quadrant, reflecting diluted associations due to widespread clinical application; CTLA-4 hypokalemia (ROR = 3.22, N = 251) demonstrates good sample-association alignment. All LAG-3 cases are in the low-risk area, and the ROR was lower than 2. The sample size was small.

Figure 1D priority matrix integrates four-dimensional scoring (Cecco et al., 2024). PD-L1 hypokalemia scores 4 points. It is classified as medium-to-high priority. Clinical relevance scores 1 point. Reporting rate scores 0 points (less than 1%). Association stability scores 2 points (all 4 algorithms yield positive results). Mortality scores 1 point (25.7%). Mortality is higher for hypomagnesemia (32.1%). Its clinical relevance is low (did not meet the criteria for IME). PD-1 hypokalemia scores 3 points. CTLA-4 hypokalemia also receives 3 points. It is classified as a moderate priority. The priority score is 6–8 points. It is classified as a high priority. But no event reaches the high-priority threshold in this study. Electrolyte disturbances could be managed through monitoring. Immediate discontinuation of treatment is not required.

### 3.5 Subgroup risk variation and identification of high-risk populations

We conduct a subgroup analysis. It identifies risk modifiers across three categories. Please refer to the forest plot (Figure 5). The blue data points represent comparisons between severe and non-severe outcomes. The orange points represent comparisons between men and women. The green points represent comparisons between fatal and non-fatal outcomes.

**FIGURE 5:**
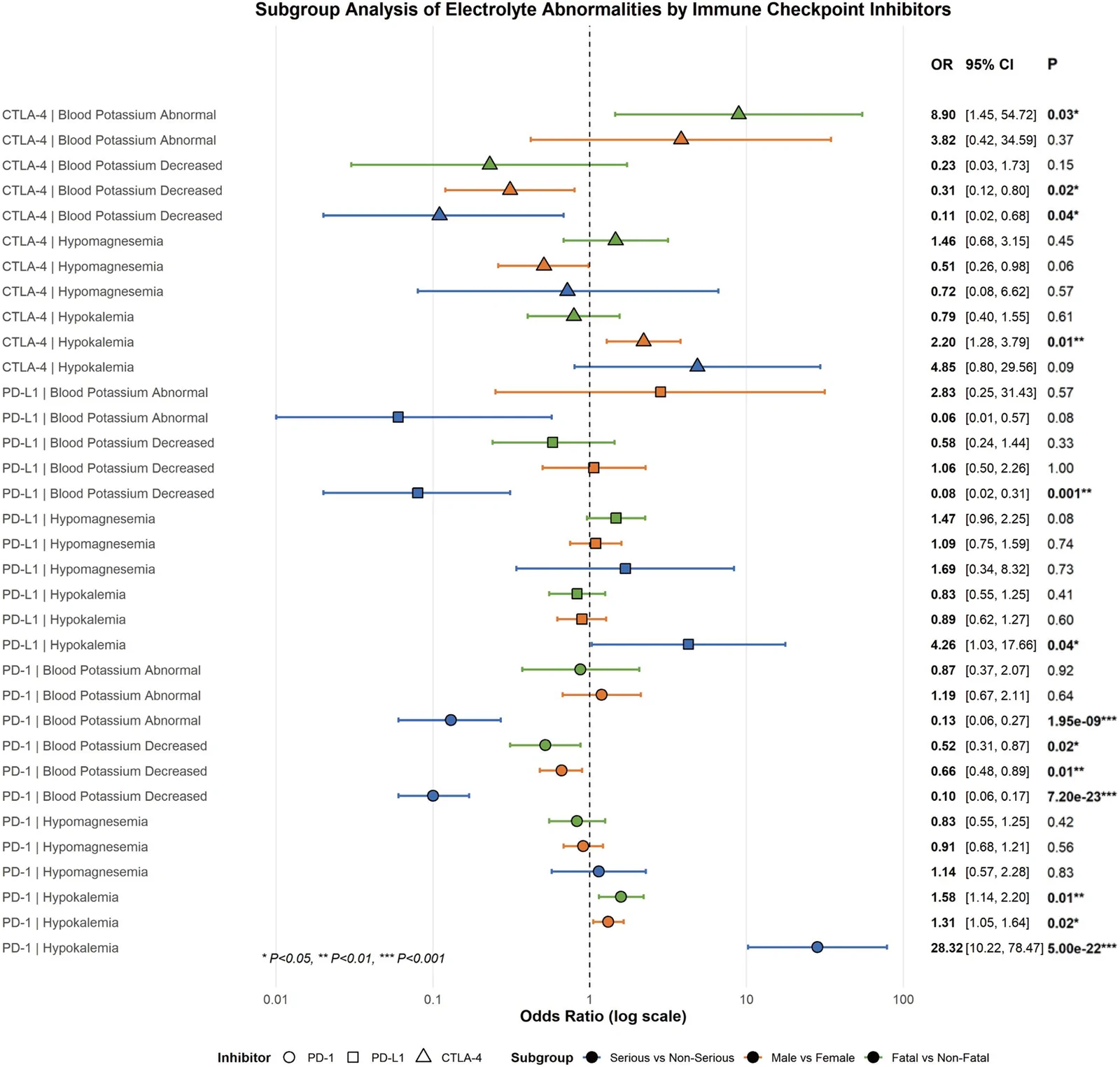
Subgroup Analysis of immune checkpoint inhibitor (ICI)-Associated Electrolyte Abnormality Risk by Patient and Clinical Characteristics (FAERS Database) Forest plot displaying odds ratios (OR) and 95% confidence intervals for subgroup analyses of electrolyte abnormalities (hypokalemia, hypomagnesemia, blood potassium decreased, blood potassium abnormal) associated with different ICIs (PD-1, PD-L1, CTLA-4). The X-axis represents odds ratio (log scale), and the Y-axis is stratified by drug and adverse event type. Shape symbols indicate ICI type: circle = PD-1 inhibitors, square = PD-L1 inhibitors, triangle = CTLA-4 inhibitors; color coding indicates subgroup type: blue = serious vs. non-serious adverse events, orange = male vs. female, green = fatal vs. non-fatal events. OR values and P-values are labeled on the right, with significance levels: P < 0.05, P < 0.01, P < 0.001. Vertical dashed line indicates OR = 1 (no effect). Key findings: PD-1 inhibitor-associated hypokalemia showed significantly elevated risk in the serious adverse event group (OR = 28.32, 95% CI: 10.22–78.47, P = 5.00 × 10^−22^); CTLA-4 inhibitor-associated hypokalemia showed significantly higher risk in male patients compared to females (OR = 2.20, 95% CI: 1.28–3.79, P = 0.01). LAG-3 inhibitors were not included due to insufficient sample size.

For serious adverse events, the risk of PD-1 inhibitor-related hypokalemia is significantly increased (Odds Ratio [OR] = 28.32, 95% CI: 10.22–78.47, P = 5.00 × 10^−22^). This indicates disease severity as a key risk amplifier for PD-1 inhibitors. The gender subgroup requires consideration. The risk of male CTLA-4 users is higher than that of female users (OR = 2.20, with a 95% confidence interval of 1.28–3.79, and a P value of 0.01). This represents a 120% increase in risk. For fatal versus non-fatal outcomes, we observe a significant difference in hypokalemia associated with PD-1 inhibitors (OR = 1.58, P = 0.01). We also observe a difference in hypomagnesemia associated with PD-L1 inhibitors (OR = 1.31, P = 0.02). No P-values indicated a significant difference for CTLA-4 inhibitors.

Notably, Figure 5 reveals opposite subgroup association patterns across different preferred terms (PTs). For hypokalemia, PD-1 inhibitors show significantly increased risk in the fatal (P = 0.008), male (P = 0.022), and severe (OR = 28.32, P < 0.001) subgroups. In contrast, for blood potassium decreased, the direction of subgroup associations is entirely opposite: risk is higher in the non-fatal subgroup than in the fatal subgroup, higher in females than in males (PD-1/CTLA-4), and higher in the non-severe subgroup than in the severe subgroup. PD-L1 inhibitor-associated blood potassium decrease also shows marginal significance (P = 0.08) in the severe versus non-severe subgroups.

### 3.6 Nonlinear association between hypokalemia time-to-onset and mortality risk

RCS analysis shows that there is a significant nonlinear relationship between onset time and death risk. In Figure 6A, the death risk of immune checkpoint inhibitor-related hypokalemia has an asymmetric U-shaped relationship with onset time, and the P-value of the nonlinear test was less than 0.001. A total of 979 reports of hypokalemia with complete time data were included in the FAERS database, of which 199 were CTLA-4-related, 470 were PD-1-related, 308 were PD-L1-related, and 2 were LAG-3-related; 157 of these were death cases. The curve is standardized to the true nadir at 60 days, where the hazard ratio (HR) for the lowest death risk was equal to 1. Early-onset (<30 days) hypokalemia shows a markedly elevated mortality risk, with a peak HR of approximately 1.9 and an upper 95% CI bound of approximately 3.2, declining steeply to the nadir at 60 days. Late-onset (>90 days) hypokalemia shows a gradually increasing mortality risk, with HR ranging from approximately 1.0–1.8 between 90 and 350 days, forming an asymmetric U-shaped “biphasic high-risk” pattern accompanied by a prolonged late-risk phase.

**FIGURE 6:**
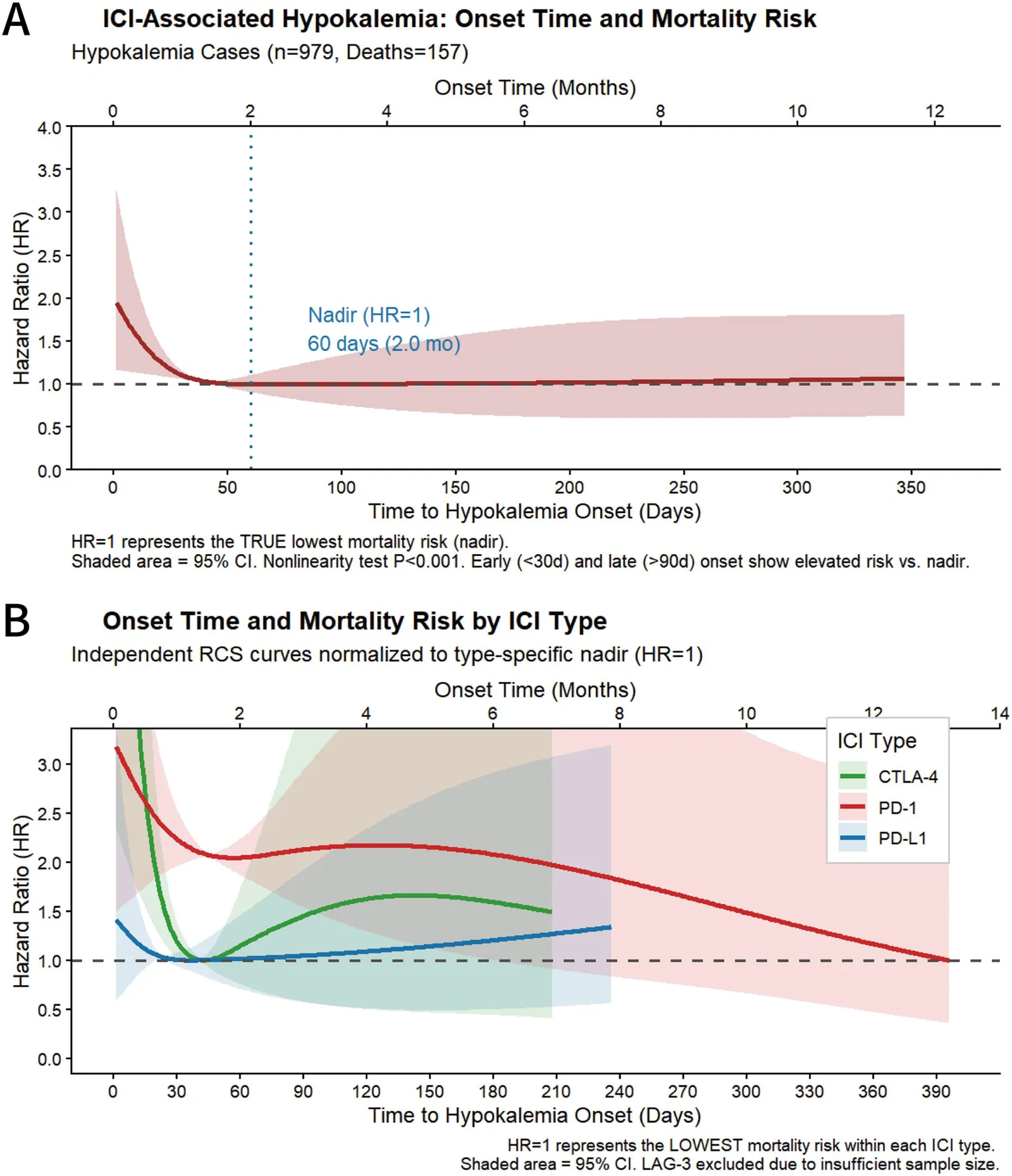
Nonlinear Association Between Time-to-Onset of Hypokalemia and Mortality Risk in immune checkpoint inhibitor (ICI)-Treated Patients (FAERS Database). **(A)** Overall Restricted Cubic Spline Analysis. RCS Cox model showing the nonlinear relationship between hypokalemia time-to-onset (days, X-axis) and hazard ratio for death (HR, Y-axis) across all ICI types combined. The curve is normalized to the true nadir (lowest mortality risk, HR = 1) at 60 days (2.0 months). The reddish-brown curve represents the fitted restricted cubic spline, with shaded area indicating 95% confidence interval. Early-onset (<30 days) hypokalemia shows markedly elevated mortality risk (HR peak ∼1.9, 95% CI upper bound ∼3.2), declining steeply to reach the nadir at 60 days. Late-onset (>90 days) hypokalemia shows gradually increasing mortality risk (HR ∼1.0–1.8 from 90 to 350 days), forming an asymmetric U-shaped “biphasic high-risk” pattern with a prolonged late-risk phase. Nonlinearity test P < 0.001. Analysis included 979 reports, with 157 deaths. **(B)** Stratified Analysis by ICI Type. Independent restricted cubic spline curves for CTLA-4 inhibitors (green), PD-1 inhibitors (red), and PD-L1 inhibitors (blue), each normalized to its type-specific nadir (HR = 1). Shaded areas represent 95% confidence intervals. Three distinct temporal patterns emerge: CTLA-4 inhibitors exhibit an “acute high-risk with delayed rebound” pattern, with extreme early HR > 3.0, nadir at ∼30 days, and progressive risk elevation to HR∼1.5 by 240 days without a sustained safety window. PD-1 inhibitors demonstrate a “progressively protective” pattern, with HR decreasing from ∼3.2 to ∼2.1 by 60 days, maintaining a mid-term plateau (HR ∼2.1–2.2 at 120–240 days), and approaching nadir (HR = 1) at 390 days, indicating improved prognosis when hypokalemia occurs later. PD-L1 inhibitors show an “early low, late elevated” pattern, with rapid decline to nadir at ∼30 days (HR = 1) followed by gradual risk increase to HR∼1.3 by 240 days. Note: Beyond 300 days, the number of at-risk cases decreases substantially (PD-1: n < 50; PD-L1: n < 30; CTLA-4: n < 20), and late-phase HR estimates should be interpreted with caution due to widening confidence intervals from sparse reporting. LAG-3 inhibitors were excluded due to insufficient sample size (n = 2).

Figure 6B is stratified by ICI type. Due to insufficient sample size (only 2 cases with calculable time data), no separate risk curve is plotted for LAG-3 inhibitors. Independent restricted cubic spline curves are fitted for CTLA-4 (green), PD-1 (red), and PD-L1 (blue) inhibitors, each normalized to its type-specific nadir (HR = 1). Shaded areas represent 95% confidence intervals. Three distinct temporal patterns emerge: CTLA-4 inhibitors exhibit an “acute high-risk with delayed rebound” pattern, with extreme early HR > 3.0, rapid decline to nadir at ∼30 days (HR = 1), and subsequent rebound to HR∼1.5 by 240 days without a sustained safety window. PD-1 inhibitors show a “progressively protective” pattern, with HR decreasing from ∼3.2 at onset to ∼2.1 by 60 days, maintaining a mid-term plateau (HR ∼2.1–2.2 at 120–240 days), and approaching the type-specific nadir (HR = 1) only at the late observation endpoint of 390 days, suggesting a prolonged risk attenuation period. PD-L1 inhibitors display an “early low, late elevated” pattern, with rapid decline to nadir at ∼30 days (HR = 1) followed by gradual risk increase to HR∼1.3 by 240 days, presenting the most stable overall risk profile but lacking a protective period.

## 4 Discussion

This study combines cross-database pharmacovigilance signals and nonlinear temporal risk modeling to construct a target-specific inflammatory pharmacovigilance framework for ICI-related hypokalemia. PD-L1 inhibitors show the strongest electrolyte disruption signal (ROR = 3.20), are characterized by ultra-early onset (median 23 days) and the highest case fatality rate (23.7%), a pattern consistent with the hypothesis that acute T-cell-mediated tubular injury—driven by constitutive PD-L1 expression on renal tubular epithelial cells (Sprangers et al., 2022; Tian et al., 2022) —may underlie the ultra-early onset and elevated case fatality rate. This mechanistic attribution is inferred from published tissue expression data rather than directly established by the pharmacovigilance database. In contrast, CTLA-4 inhibitors displayed a biphasic temporal pattern reflecting delayed hypothalamic-pituitary-adrenal (HPA) axis inflammation (Cui et al., 2022; Faje et al., 2014), while PD-1 inhibitors showed progressive risk attenuation suggesting indirect immune-mediated nephrotoxicity (Moturi et al., 2024). Additionally, the observed male predominance in CTLA-4–associated hypokalemia (OR = 2.20) aligns with the established sexual dimorphism in immune checkpoint inhibitor toxicities, including the higher incidence of ipilimumab-induced hypophysitis in male patients (Faje et al., 2014), although sex-specific reporting biases cannot be excluded. The distributions at different moments show different pharmacodynamic characteristics of inflammation, not unified off-target toxicity.

The divergent signal strength and mortality profiles between PD-L1 and PD-1 inhibitors may reflect fundamental differences in non-tumor tissue biology. PD-L1 is constitutively expressed on renal tubular epithelial cells, predisposing to direct T-cell-mediated tubular injury and cytokine-driven electrolyte wasting upon blockade (Sprangers et al., 2022; Tian et al., 2022). In contrast, PD-1 is predominantly expressed on immune cells, and its blockade may induce hypokalemia indirectly via systemic inflammatory nephrotoxicity (Moturi et al., 2024), potentially explaining the attenuated early signal and the prolonged risk attenuation pattern. Additionally, differences in antibody binding kinetics and Fc-effector functions between PD-L1 and PD-1 agents may contribute to the divergent temporal toxicity profiles, although direct comparative pharmacokinetic data are currently lacking.

This study exemplifies a computational pharmacovigilance paradigm. The ensemble signal detection strategy mirrors machine learning bagging methodologies, where cross-algorithm consensus reduces individual algorithmic bias. The RCS Cox framework addresses a critical gap in conventional pharmacovigilance—its inability to model nonlinear temporal dynamics—by leveraging spline-based nonlinear basis expansions, a technique widely adopted in modern predictive modeling. Furthermore, the four-dimensional priority algorithm provides clinical interpretability via explicit rule-based prioritization, ensuring transparency without *post hoc* algorithmic explainability techniques. Conceptually aligned with the explainable AI movement, this scoring framework remains a clinically adapted decision tool (Cecco et al., 2024) rather than a machine-learning-derived interpretability method.

From the dimension of inflammatory pharmacology, the neuromuscular and neuroendocrine implications of these temporal patterns warrant particular attention. We hypothesize that severe hypokalemia may adversely impact skeletal muscle function via reduced Na^+^/K^+^-ATPase activity and compromised acetylcholine release at the neuromuscular junction (Liu et al., 2024), potentially establishing a clinically relevant muscle-immune metabolic interplay in ICI-treated patients. This hypothetical axis remains to be prospectively validated through integrated muscle strength grading and electromyography in electronic health record cohorts. Concomitant hypomagnesemia—especially PD-L1-associated subtype with 32.1% case fatality—further impairs renal potassium reabsorption and neuromuscular transmission, establishing a self-perpetuating cycle of electrolyte-driven inflammatory dysfunction (Floris et al., 2025). Neuroendocrinologically, HPA axis disruption from CTLA-4-induced hypophysitis represents cortisol signaling dysregulation (Hori et al., 2024), where hypokalemia may compound both physical fatigue and psychological distress prevalent in 24.3% of long-term ICI survivors (Sahin et al., 2025). These research findings identify electrolyte disturbances not merely as metabolic complications but as modifiable drivers of inflammatory neuromuscular and neuroendocrine pathology.

At present, the SITC and ESMO guidelines give suggestions that, in the absence of target-specific temporal guidance, unified laboratory monitoring should be carried out (Puzanov et al., 2017; Haanen et al., 2022). The pharmacovigilance-derived framework we constructed involves inflammation pharmacology-adjusted monitoring: within weeks 0–2, we focus on intensive electrolyte monitoring for PD-L1 inhibitors because of the acute tubular inflammatory surge; continuous monitoring for CTLA-4 inhibitors is carried out because delayed HPA axis inflammation leads to loss of safety windows; and for PD-1 inhibitors, after initial intensive monitoring within weeks 0–4, the monitoring interval is extended after 2 months. For patients receiving ICI treatment, it is proposed to maintain serum potassium in the range of 3.8–4.0 mmol/L. Before potassium adjustment, magnesium should be evaluated and corrected. This threshold is extrapolated from critical care evidence (Goyal et al., 2012; Kraft et al., 2005), is not established as an ICI-specific standard, and prospective validation is still needed in oncology populations. No events reached the high-priority discontinuation threshold (6–8 points), allowing continued ICI therapy with active electrolyte correction rather than reflexive treatment interruption.

Although this study relies on clinical-level real-world data, the target-specific temporal signatures identified here generate falsifiable hypotheses for future multi-omics integration. For instance, the ultra-early PD-L1 pattern, the biphasic CTLA-4 trajectory, and the progressive PD-1 attenuation each imply distinct inflammatory mechanisms that could be validated through integrated transcriptomic and metabolomic profiling. We posit that our computational framework provides the temporal and target-specific scaffold upon which future AI-driven multi-omics studies can anchor mechanistic validation (Selevsek et al., 2020). The existing limitations include retrospective underreporting, missing denominator data, and a small number of LAG-3 samples. Statistical associations cannot establish causal relationships, and survival analysis does not take into account competing mortality risks. Additionally, FAERS and VigiBase employ distinct case identification systems without shared global identifiers, precluding formal deduplication between databases. Potential overlap in spontaneously reported cases may therefore exist, which would attenuate cross-database heterogeneity and introduce conservative bias rather than inflate false-positive signals. The database lacks direct neurological outcome indicators; the proposed neuromuscular connection remains hypothetical and needs to be prospectively verified in electronic health record cohorts through integrated muscle strength grading and endocrine function testing. Nevertheless, the cross-database design, cross-validation of the four algorithms, and flexible RCS modeling provide robust real-world evidence for precision inflammatory pharmacovigilance.

## 5 Conclusion

This study establishes a translational interpretable multi-algorithm ensemble framework to resolve target-specific temporal risk patterns of immune checkpoint inhibitor-associated hypokalemia from multi-source real-world data. PD-L1 inhibitors demonstrated the strongest signal, with an ROR of 3.20 (95% CI: 2.95–3.48), ultra-early onset (median 23 days), and the highest case fatality rate (23.7%). Nonlinear survival analysis identified an asymmetric biphasic mortality pattern with a nadir at 60 days: early-onset events (≤30 days) peaked at HR ∼1.9, while late-onset events (>90 days) gradually rose to HR ∼1.8 within 350 days. By bridging computational signal detection with target-specific clinical monitoring protocols, this work exemplifies a translational pharmacology paradigm for immune-oncology drug safety. These computational signatures reflect distinct target-specific inflammatory pharmacologies, provide a computationally grounded translational foundation for precision electrolyte monitoring, and generate falsifiable hypotheses for future multi-omics mechanistic validation.

## Data Availability

The original contributions presented in the study are included in the article/supplementary material, further inquiries can be directed to the corresponding author.
